# Visual outcomes of cataract surgery: An observational study of ten years from a tertiary eye care hospital in Pakistan

**DOI:** 10.12669/pjms.37.7.4428

**Published:** 2021

**Authors:** Shahid Ahsan, Muhammad Saleh Memon, Sadia Bukhari, Tauseef Mahmood, Muhammad Faisal Fahim, Uzma Haseeb, Muhammad Arslan

**Affiliations:** 1 Dr. Shahid Ahsan, MPhil (Bio), MPhil (NCD), PhD fellow Department of Biochemistry, Jinnah Medical & Dental College, Karachi, Pakistan; 2 Dr. Muhammad Saleh Memon, FRCS(Eden) Department of Research, Al Ibrahim Eye Hospital, Isra Postgraduate Institute of Ophthalmology, Karachi, Pakistan; 3 Dr. Sadia Bukhari (MS Ophth) Department of Ophthalmology, Al Ibrahim Eye Hospital, Isra Postgraduate Institute of Ophthalmology, Karachi, Pakistan; 4 Mr. Tauseef Mahmood, M.Sc. (Statistics) Department of Research, Al Ibrahim Eye Hospital, Isra Postgraduate Institute of Ophthalmology, Karachi, Pakistan; 5 Mr. Muhammad Faisal Fahim, M.Sc. (Statistics) Department of Physical Therapy, Bahria University Medical & Dental College, Karachi, Pakistan; 6 Dr. Uzma Haseeb (FCPS) Department of Ophthalmology, Al Ibrahim Eye Hospital, Isra Postgraduate Institute of Ophthalmology, Karachi, Pakistan; 7 Mr. Muhammad Arslan (MCSW) Department of Research & Excellence, Al-Tibri Medical College, Karachi, Pakistan

**Keywords:** Cataract Extraction, Ophthalmic diseases burden, Visual Acuity, Phacoemulsification

## Abstract

**Objectives:**

To observe and analyze the visual outcomes of cataract surgery of ten years at a tertiary eye care hospital, Karachi.

**Methods:**

An observational study with retrospective data search was conducted in Al Ibrahim Eye Hospital (AIEH), Karachi. Data of all adults (above 16 years) who underwent cataract surgery from 2010-2019 was retrieved from HIMS. Presence of opacity in the lens was labelled as cataract. Surgery was advised when patient’s BCVA was found to be ≤ 6/18. Visual assessments of the patients were done on day 01, one week and six weeks postoperatively. Postoperatively, 6/6 – 6/12 was considered as good, 6/18 as mild visual impairment, < 6/18 to 6/60 as moderate visual impairment and < 6/60 as severe visual impairment.

**Results:**

A total of 1,027,840 patients visited AIEH with different eye diseases. Among 1027840 individuals, cataract was identified in 88443 (8.6%). Surgery was advised to 58371 and performed in 38616. Records of operated cases (38616) were retrieved. Mean age of patients was 54.96±14.22 years. There were 20578 (53.29%) males and 18038 (46.71%) females who underwent surgery . At the end of sixth week, 3561 (18.4%), patients were found to have “good vision”, 12242 (63.43%) had mild visual impairment, 2676 (13.86%) had moderate visual impairment and 822 (4.26%) had severe visual impairment. Corneal Complications was the commonest cause (33.56%) at sixth week.

**Conclusions:**

The institution achieved WHO recommended criteria of “good visual outcome” (6/6 to 6/18) of 81.83% which is near to recommended ≥ 90% and severe visual impairment of 4.26%.

## INTRODUCTION

Cataract is the gradual cloudiness of the transparent ocular lens. It is regarded as a single largest cause of reversible blindness worldwide. Nearly 45% of global blindness is attributed to cataract, accounting 15 million out of 33.6 million cases of the global blindness.^1^ Every year nearly one million people worldwide became blind due to cataract expected to raise further due to population growth and longer life expectancy.^2^

The burden of vision loss in Pakistan had been on the rise since 1990 and is estimated to increase at a steady rate by 2025. Pakistan ranked third, among south Asian countries, after India and Bangladesh in the prevalence of blindness and vision impairment. Presbyopia, refractory disorder and cataract contributed >90% cases of blindness and severe vision loss. Nearly >50% of these cases in the country were attributed to cataract.^2^

Cataract is primarily an aging phenomenon that cannot be prevented. However, vision impairment by cataract can be restored by surgery. Though cataract surgery is the commonest procedure performed by the ophthalmic surgeons, substantial variations in cataract surgery rate (CSR) between countries were observed. Cataract surgery rate is used as a proxy indicator of access to cataract services in a country.^2^ In 2004, WHO mapped Pakistan with other countries of Eastern Mediterranean Region having 2000-3000 CSR. A dramatic rise in CSR over in the last 14 years in the country was observed touching to >5000 cases in 2018. This rise in rate of cataract surgery is likely be due to increased access to eye care facilities, increased aging population and reduced thresholds of visual impairment (from 6/60 to 6/12) as an indication for surgery. For example, in Australia reduction in visual impairment threshold from 6/60 to 6/12 as an indication for cataract surgery resulted in nearly fivefold increase in number of people eligible for cataract surgery.^3^ Moreover, with introduction of new techniques and technologies for cataract raises the demand and patient’s expectations for better vision. These facts raise the concerns about the quality of outcomes and concept of surgical audit. In order to achieve good results, it is imperative that surgeons and eye care centers should audit their performance regularly.^4^

Present study has followed the new recommendations^5^. In this study, authors audited the 10 years results of cataract surgery at Al- Ibrahim Eye Hospital, Karachi, which is tertiary level eye care center draining sub urban and rural area of Karachi and surrounding districts of Sindh and Baluchistan.

## METHODS

It was an observational study with retrospective data retrieved from HIMS for the year 2010-2019 carried out at Al Ibrahim Eye Hospital, a tertiary eye care center, Karachi. Data of all adults (above 16 years) undergoing cataract surgery by Extra-capsular cataract extraction (ECCE), phacoemulsification with IOL (Phaco + IOL) implant and Intra capsular cataract extraction (ICCE) with or without IOL were included in the study. Missing records, dropped follow-ups, cataract surgery combined with other ocular surgery like trabeculectomy, vitrectomy, keratoplasty, complicated and secondary cataract were excluded.

All patients visiting the out patient’s department of Al Ibrahim Eye Hospital for ophthalmic consultation, underwent detail ophthalmic examination like Best corrected visual acuity (BCVA), Slit lamp examination, IOP and Fundus examination. Presence of opacity in the lens was labelled as cataract. B-scan was performed in patients whose fundus was not visible with 90 D fundus examination. Surgery was advised when patient’s BCVA found to be < 6/18 or patient was not satisfied with his present vision. Pre-operative investigation included Hepatitis B, C, HIV and Random Blood Sugar level. Keratometry and biometry was done to determine IOL number. The choice of procedure was based on type and grade of cataract. Extracapsular extraction was done in mature and hyper mature cataracts where phacoemulsification was problematic. IOL was implanted in both procedures. Post-operatively topical antibiotics and steroids were prescribed to all patients. First follow-up was scheduled the next day of surgery. Subsequent follow-up was at 1^st^ week and 6^th^ week after surgery. BCVA was recorded at all visits. Visual acuity (BCVA) was graded according to new recommendations where visual outcome is categorized into 4 grades: good 6/6 to 6/12, minimal visual impairment 6/18, moderate visual impairment < 6/18 to 6/60 and severe visual impairment < 6/60.

WHO recommends following categories and levels to be achieved:^6^

**Table:** 

*Category*	*Visual range*	*Available correction*	*Best corrected VA*
Good	6/6 -----6/18	>85%	>90%
Borderline	<6/18 …..6/60	< 10%	<5%
Poor	<6/60	<5%	<5%

Almost all national studies^7^-^13^ followed this classification to report surgical outcome. As the cutoff point for surgery has been revised from 6/60 to 6/18 or even 6/12,^14^ the visual outcomes need to be revised. The new recommendations^5^ consider 6/6 – 6/12 as good, 6/18 as mild visual impairment, < 6/18 to 6/60 as moderate visual impairment and < 6/60 as severe visual impairment.

### Statistical Analysis

Data was retrieved from Hospital Information Management System (HIMS) and exported to SPSS version 23.0. Mean and Standard deviation was calculated for continuous variable like age. Frequencies and percentages were reported for categorical variables like age groups, gender, type of surgery, categories for visual acuity and causes of reduced BCVA. Chi-square test was applied to see the significance. P-value ≤ 0.05 considered to be statistically significant.

### Ethical Approval

(Ref: REC/IPIO/2020/031, Dated: September 16, 2020)

## RESULTS

**Fig.1:**
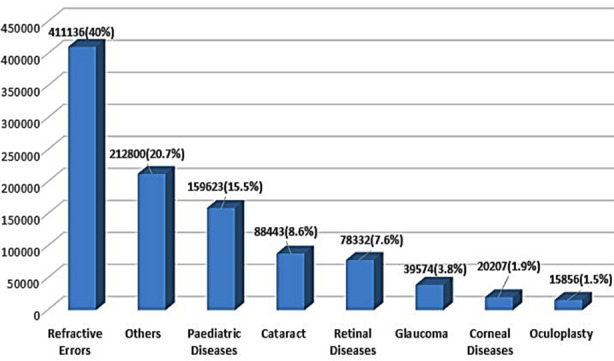
People attending Outpatient department (2010-2019).

**Fig.2:**
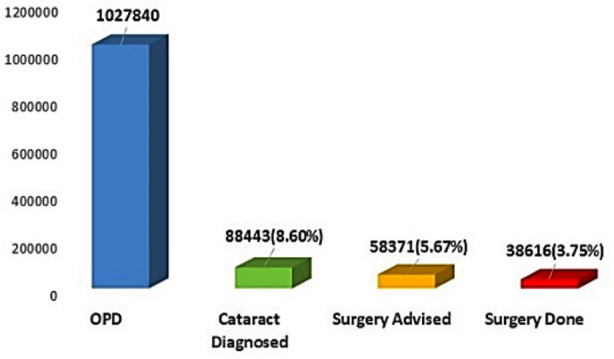
Cataract Burden and Coverage from 2010 to 2019.

During the year 2010-2019, total of 1,027,840 patients visited Al- Ibrahim Eye Hospital with different eye problems. Among them 88443 (8.6%) individuals were labelled as cataract, Fig.1. Number of cataracts which met the criteria of surgery were 58371 and the number of cataracts operated were 38616. Details shown in Fig.2.

Age of patients ranged from 16 to more than 60 years. Mean age of patients operated was 54.96±14.22 years. An increasing frequency of age group was seen from 46 to 60 years. Gender distribution was observed as male 20578 (53.29%) and female 18038 (46.71%). Phacoemulsification with PC IOL (Phaco + PC IOL) was done in 33990 (88.02%) and Extracapsular Cataract extraction with Posterior Chamber Intraocular Lens (ECCE + PC IOL) was done in 3684 (9.54%) patients. Remaining individuals 942 (2.4%) had Intra capsular cataract extraction (ICCE) with or without IOL (Table-I).

**Table I:** Demographic Characteristics of patients.

*Age Group*	*Frequency (%) (n=38616)*
16-30	7.35%
31-45	13.27%
46-60	49.14%
>60	30.24%
Gender	
Male	20578 (53.29%)
Female	18038 (46.71%)
Type of Surgery	
(PHACO + PC IOL) Phacoemulsification plus Posterior Chamber Intraocular Lens	33990 (88.02%)
(ECCE + PC IOL) Extracapsular Cataract extraction plus Posterior Chamber Intraocular Lens	3684 (9.54%)
Others	942 (2.44%)

### Results at 6 week follow up

Total number completing follow up of 6 weeks were 19301 (50%) Those with “good vision” were 3561 (18.4%), with mild visual impairment were 12242 (63.43%), with moderate visual impairment were 13.86% and those with sever vision impairment were 4.26% (Table-II).

**Table II:** Visual Acuity from Baseline (pre-operative) to Sixth Week Post-Operative.

*Visual Acuity*	*Pre-Operative*		*1st day Post-Op*		*1st Week Post-Op*		*6th week Post-Op*	

	*Frequency*	*Percent*	*Frequency*	*Percent*	*Frequency*	*Percent*	*Frequency*	*Percent*
6/6 – 6/12 (Good Vision)	2678	6.93	4281	12.3	4936	17.61	3561	18.45
6/18 (Mild Vision Impairment)	6211	16.08	17120	49.17	14747	52.62	12242	63.43
< 6/18 – 6/60 (Moderate Vision Impairment)	18002	46.62	10233	29.39	6734	24.03	2676	13.86
< 6/60 (Severe Vision Impairment)	11725	30.36	3181	9.14	1611	5.75	822	4.26
Total	38616	100	34815	100	28028	100	19301	100

Drop outs were 9.84% (3801) patients on 1^st^ postoperative day, 27.41% (10588) on 2^nd^ follow up at one week and 50% (19315) at 6^th^ week. At 6 week follow up, 18.27 % of the Phaco IOL had “ good vision”, 65.04% had mild visual impairment, 13.03% had moderate and only 3.64% had severe visual impairment. Respective results with ECCE IOL were 19.2%, 55.7%, 17.78% and 7.18%. Severe visual impairment with Phaco is 3.64%, which is well within recommended level of < 5%; but 7.18% with severe visual impairment in ECCE IOL is more than desirable (Table-III).

**Table III:** Type of Surgery and Visual Outcomes at Sixth Week Follow-up.

*BCVA Categories*	*Type of Surgery*		*P-value*

	*ECCE + PC IOL n=3356 (17. 38%)*	*Phaco + PC IOL n=15945 (82.6%)*	
6/6 - 6/12 (Good Vision)	647 (19.2%)	2914 (18.2%)	0.347
6/18 (Mild Vision Impairment)	1871 (55.7%)	10371 (65%)	
< 6/18 - 6/60 (Moderate Vision Impairment)	597 (17.8%)	2079 (13.03%)	
< 6/60 (Severe Vision Impairment)	241 (7.1%)	581 (3.6%)	
Total	3356	15945	

**Fig.3:**
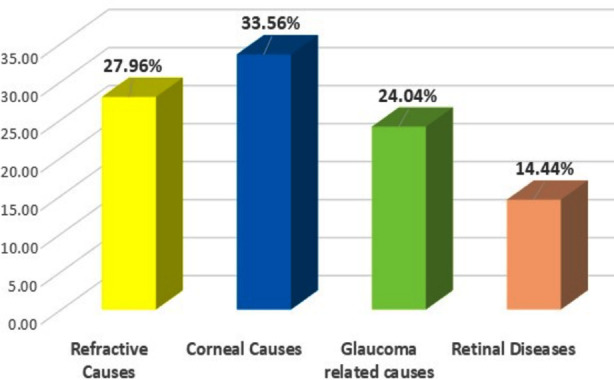
Causes of BCVA <6/18 at Sixth Week Follow-up.

Causes of BCVA< 6/18 at 6^th^ week follow-up is shown in (Fig.3): Main causes of reduced vision were related to cornea (33.56%) and refractive errors (27.96%), while others were due to associated causes like glaucoma (24.04%) and retinal diseases (14.44%).

## DISCUSSION

Cataract surgery is one of the most frequently performed surgical procedure worldwide. There is no guideline from international organization about visual acuity threshold for cataract surgery. Ideally any patient who has symptoms of cataract should offer surgery. Priority however should be given to the people who are blind from un-operated cataract as this segment of patient benefitted at most from surgery. This trend is however changed in current era because patients’ needs and the results that they expect to obtain from surgery have changed. Thus patients in early stages of the development of cataract and with visual acuity of 6/6 to 6/12 opted to undergo cataract surgery often to correct the refractive error rather than to reverse visual impairment. National studies from Pakistan also reported significant proportion of patient undergo cataract surgery at good VA of 6/6- 6/18. A study from Aga Khan reported^7^ 57.9% of patients that underwent cataract surgery had good pre-operative VA of 6/6-6/18. In the present study 23.01% had pre-operative VA of > 6/18 , 6.93% patients with VA (6/6- 6/12) and 16.08% patient had mild impairment (VA 6/18). Seventy-seven percent (77%) patients, had moderate and severe preoperative visual impairment (VA <6/18). Presence of higher frequency of poor preoperative VA is an indication of inadequate provision of cataract surgical services. In contrast induction of patients with better VA resulted in rise in CSR without having the influence on reduction of blindness. This is particularly observed in low- and middle-income countries where despite of increased cataract surgical rate prevalence of blindness is also on rise. According to a report of WHO^15^ nearly two decades earlier most people in India and Kenya had VA of < 3/60 before undergoing cataract surgery. Though recently there is dramatic increase in the number of cataract operations, it had no impact on the number of people who are blind from cataract.

As regards gender, in developed countries, females accounted for 59%-66% of all cataract surgeries.^16^ Whereas in developing countries gender bias is known to exist with more men than women had cataract surgery. Since women have higher incidence of cataract and longer life expectancy, in order to have equal access to care for cataract nearly 60-70% of the individuals undergoing cataract surgery must comprise of women.^17^,^18^

In present study similar gender pattern was observed with more men compared to women (56.46 % vs 43.54%) undergone cataract surgery, which is 12.92% lower in women. Various studies from Pakistan reported Cataract surgical coverage (CSC) of 69.9 – 72.2% among men while reported CSC for women was 39.6 – 60.2 %. Studies also reported gross gender inequity in terms of CSC from other countries of South Asia. Like in India CSC was found to be 27% higher in men than women. Nearly 2/3^rd^ of the global burden of blindness was found in women, a higher rate of cataract surgery in female populations should be emphasized in cataract programs.^16^

Reporting the outcome of cataract surgery is important in order to establish the bench mark and to audit the performance of an institute. Nearly 19755 (33.9%) individuals advised for surgery did not avail intervention. It remains to be explored why 33.9% people with cataract did not avail surgery although being non-profit organization, surgery is offered at very subsidized rate. These patients availed the free consultation; but did not avail the intervention. Second observation in this study was patient dropout rate in follow ups after cataract surgery. Our findings indicated that people seek and receive cataract surgery but miss out on regular follow-up consultations. Most likely reason of both these occurrences is financial constraint. Though being charitable institute services in this center is offered at very subsidize rate, for the very poor segment of people it remains out of pocket. People were too poor to afford even subsidized cost and come for follow up. There exists strong relationship of poverty with cataract surgical rate (CSR), and postoperative visual acuity.^8^ Study on “relationship of output, outcomes of cataract surgery and national indices of socioeconomic development” has shown that good vision outcomes was lowest in Pakistan (6%) and highest in Malaysia (86%), however author did not cite the source of this information.^19^

Over the period of ten years, change in the operation procedure was observed from ECCE (17.38%) to Phacoemulsification (82.6%). ECCE has been replaced by Phaco surgery except in very hard cataracts. Our main worry is moderate vision impairment (<6/18 to 6/60) of 13.03% with Phaco surgery.

Our results are comparable with national and afro Asian countries. Most of these studies followed the old recommendations where “good vision” includes 6/6 to 6/18, border line 6/24-6/60 and poor vision <6/60. In our study if 6/18 is considered as “good vision”, the number will be 81.88% with Phaco and 74.9% with ECCE IOL. Report from university college hospital in IBADAN showed “Good vision” in 78.8%, borderline vision in 17.4%, and severe visual impairment in 3.8% after cataract surgery.^20^ A study from Bangladesh also showed that at final follow-up visit (6 to 8 weeks), best corrected visual acuity (BCVA) was good (≥6/18) in 68.5%, borderline in 20.1% and poor in 11.5%.^21^


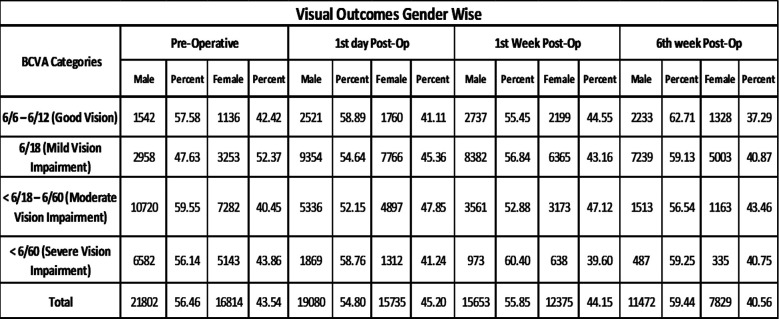


Rapid assessment of cataract surgical services in Malakand district showed good, borderline and poor visual acuity of 40.5%, 22% and 14.3% respectively.^9^ Malik and colleagues^10^ demonstrated that visual outcome after 8 weeks post operatively good visual outcome in 90% or more of operated eyes. A study from a private sector reported^15^ overall, 93.3% of the operated eyes had good visual outcome, while 4.4% and 2.2% had borderline and poor outcomes. Marie Adelaide Leprosy Centre Karachi,^11^ reported “best” corrected visual acuity in 97.2%, borderline in 2.2% and poor in 0.6% patients at >12 weeks follow up with 565 (38.7%) drop out patients. Report from Eye Unit of the Lady Reading Hospital, Peshawar, reported good Visual outcome in 88.3%, borderline in 8.3% and poor in 3.3% patients.^12^ Present study is the largest postoperative result reported nationally. Though the study has certain limitations, the outcomes of high volume of cataract surgeries reported in this study made it creditable to compare with the outcomes of cataract surgeries conducted nationally and in neighboring countries.

Like any surgical procedure, cataract surgery is also liable to complications. These complications result in poor visual outcomes and are dependent on many preoperative, intraoperative and postoperative factors. Corneal damage during surgery is the most common cause of postoperative moderate as well as severe vision impairment. This is common in the learning phase of Phaco emulsification. The second common reason for unfavorable visual outcome was due to refractive errors resulting from the surgery 13

### Limitations of the Study

Though present study is the largest study reporting post-operative outcomes of cataract, study demands carful interpretation. Deposition of data in HMIS is not real time. Data entry operator transferred data from hard copy to the data bank at a convenient time. This may result in erroneous entry which is rectified by randomly cross checking and matching the hard file entry with data bank. Nearly 33.9% people attended the hospital and diagnosed with cataract did not avail surgery in our center. Moreover, postoperative patient’s dropouts reached to 50% (19315) at 6^th^ week. We are completely unaware of the visual status of these patients. Another important aspect of this study is variability of the operating surgeon from trainee ophthalmologist to senior surgeon over 10 years.

## CONCLUSION

The institution achieved WHO recommended criteria of “good visual outcome” (6/6 to 6/18) of 81.83% which is near to recommended ≥ 90% and severe visual impairment of 4.26%.

### Authors’ Contribution:

**MSM** manuscript writing & final review.

**SA** manuscript writing, critical review & literature search.

**SB** did final review of the article.

**TM** did data collection, summarizing of data & editing of manuscript

**MFF** did Statistical analysis and result write up.

**UH** review from clinical point of view as an ophthalmologist

**MA** design of study and comments on review article.
